# Investigating the spatial variation and risk factors of childhood anaemia in four sub-Saharan African countries

**DOI:** 10.1186/s12889-020-8189-8

**Published:** 2020-01-29

**Authors:** Danielle J. Roberts, Glenda Matthews, Robert W. Snow, Temesgen Zewotir, Benn Sartorius

**Affiliations:** 1 School of Mathematics, Statistics and Computer Science, University of KwaZulu-Natal, Durban, South Africa; 2 Department of Statistics, Durban University of Technology, Durban, South Africa; 3 Population Health, Kenya Medical Research Institute-Wellcome Trust Research Programme, Nairobi, Kenya; 4 Centre for Tropical Medicine and Global Health, Nuffield Department of Clinical Medicine, University of Oxford, Oxford, United Kingdom; 5 Faculty of Infectious and Tropical Diseases, London School of Hygiene and Tropical Medicine, London, United Kingdom

**Keywords:** Adjusted odds ratio, Anaemia, Bayesian, Child, Haemoglobin level, Hierarchical geoadditive model, Spatial effect

## Abstract

**Background:**

The causes of childhood anaemia are multifactorial, interrelated and complex. Such causes vary from country to country, and within a country. Thus, strategies for anaemia control should be tailored to local conditions and take into account the specific etiology and prevalence of anaemia in a given setting and sub-population. In addition, policies and programmes for anaemia control that do not account for the spatial heterogeneity of anaemia in children may result in certain sub-populations being excluded, limiting the effectiveness of the programmes. This study investigated the demographic and socio-economic determinants as well as the spatial variation of anaemia in children aged 6 to 59 months in Kenya, Malawi, Tanzania and Uganda.

**Methods:**

The study made use of data collected from nationally representative Malaria Indicator Surveys (MIS) and Demographic and Health Surveys (DHS) conducted in all four countries between 2015 and 2017. During these surveys, all children under the age of five years old in the sampled households were tested for malaria and anaemia. A child’s anaemia status was based on the World Health Organization’s cut-off points where a child was considered anaemic if their altitude adjusted haemoglobin (Hb) level was less than 11 g/dL. The explanatory variables considered comprised of individual, household and cluster level factors, including the child’s malaria status. A multivariable hierarchical Bayesian geoadditive model was used which included a spatial effect for district of child’s residence.

**Results:**

Prevalence of childhood anaemia ranged from 36.4% to 61.9% across the four countries. Children with a positive malaria result had a significantly higher odds of anaemia [AOR = 4.401; 95% CrI: (3.979, 4.871)]. After adjusting for a child’s malaria status and other demographic, socio-economic and environmental factors, the study revealed distinct spatial variation in childhood anaemia within and between Malawi, Uganda and Tanzania. The spatial variation appeared predominantly due to unmeasured district-specific factors that do not transcend boundaries.

**Conclusions:**

Anaemia control measures in Malawi, Tanzania and Uganda need to account for internal spatial heterogeneity evident in these countries. Efforts in assessing the local district-specific causes of childhood anaemia within each country should be focused on.

## Background

Anaemia, which is a condition in which the haemoglobin (Hb) concentration is lower than that required by the body to meet its physiological needs, is a major cause of morbidity and mortality among pregnant women and young children in most Low and Middle Income countries (LMIC), particularly those in sub-Saharan Africa (SSA) [1]. Anaemia contributes to adverse health problems in children, and affects their cognitive, behavioural and physical development [2, 3]. If left untreated, the long-term effects and consequences of anaemia in early childhood are irreversible, if mortality has not occurred [3]. According to the most recent estimates of the World Health Organization (WHO), the highest anaemia prevalence of 42.6% in 2011 occurred in children under the age of five years old, which translated to just over 273 million children suffering from anaemia globally [4]. In Africa, the prevalence of anaemia in children was estimated at 62.3% in 2011 [5]. The causes of anaemia in childhood are multifactorial and interrelate in a complex way. Such causes include iron deficiency, other micronutrient deficiencies such as folate, vitamin B12 and vitamin A; intestinal parasites such as soil-transmitted helminths (STH) and Schistosoma; malaria, HIV infection, and chronic diseases such as sickle cell disease [6]. While iron deficiency is the most common cause of anaemia in high-income countries (HIC), there are many other contributing factors in LMIC. In countries that are highly malaria-endemic, particularly in SSA, malaria is a significant contributing factor to childhood anaemia [7].

While the WHO strives for goals of achieving a 50% reduction of anaemia in women of reproductive age by 2025, childhood anaemia has no such direct goals in place and thus has not received adequate attention [6]. Rather, goals for anaemia reduction in children currently coincide with Sustainable Development Goals of ending all forms of malnutrition and preventable deaths of children under 5 years of age by 2030 [8]. Furthermore, the WHO and UNICEF recommend that strategies for anaemia control be built into a country’s primary health care system and existing programmes such as maternal and child health, integrated management of childhood illness, roll-back malaria and deworming [9]. These control strategies are expected to be tailored to local conditions by taking into account the specific etiology and prevalence of anaemia in a given setting and population group. Accordingly, studies on anaemia control should be cognisant of and account for the spatial variation of anaemia in a given population. Failure to account for the spatial heterogeneity of anaemia and the possible causes of the spatial heterogeneity can result in ecological confounding and thus mislead policy makers [10].

This study investigates the spatial variation of anaemia in children aged 6 to 59 months and identifies significant risk factors associated with anaemia in these children in Kenya, Malawi, Tanzania and Uganda.

## Methods

### Study area and data

This study utilised data collected in the Demographic and Health Surveys (DHS) and Malaria Indicator Surveys (MIS) carried out in four contiguous countries in eastern sub-Saharan Africa (Additional file 1: Figure S1), namely Kenya, Malawi, mainland Tanzania and Uganda between 2015 and 2017. These include the 2015 Kenya Malaria Indicator Survey (KMIS2015), the 2017 Malawi Malaria Indicator Survey (MMIS2017), the 2015-2016 Tanzania Demographic and Health Survey and Malaria Indicator Survey (TDHS2015) and the 2016 Uganda Demographic and Health Survey (UDHS2016). The DHS and MIS were designed to provide national, regional, urban and rural estimates of key health indicators [11]. Both types of surveys followed the DHS Program’s standard procedures and methodologies. The surveys were nationally represented and utilised a stratified two-stage cluster design in which each country was stratified into their respective administrative areas and then further stratified into urban and rural areas. The first stage of sampling involved selection of the enumeration areas (EAs) or clusters from each of the urban/rural strata. In the second stage, households were systematically selected. The selected households were visited and interviewed by trained staff. A thorough review of the sampling methodology is presented in the DHS Sampling Manual [12]. Three questionnaires, namely, the household, women and men questionnaires, were carried out in the sampled households. These questionnaires were designed to collect information regarding the characteristics of the household and eligible women, men and children. In both the DHS and MIS, all children under the age of five years old in the sampled households were tested for malaria and anaemia, with the consent of a parent or guardian.

### Study variables

#### Outcome variable

In all the surveys, a child’s haemoglobin concentration was measured by finger- or heel-prick blood specimens using a portable HemoCue analyser. For this study, a binary outcome variable was used, and children with an altitude adjusted Hb level less than 11 g/dL were classified as anaemic, in accordance with the WHO definition of anaemia in children aged 6 to 59 months [13].

#### Explanatory variables

**Fig. 1:**
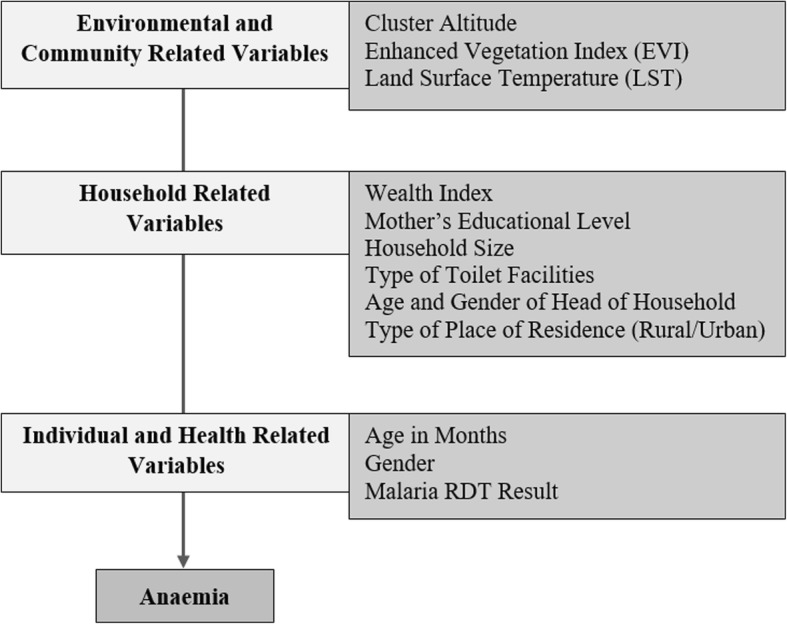
Potential risk factors of childhood anaemia considered in this study

The explanatory variables considered in this study comprised of a number of demographic, socio-economic and environmental factors (Fig. 1). Such factors included the gender and age of the child, the child’s malaria Rapid Diagnostic Test (RDT) result, the mother’s highest education level, the number of members in the household (size of the household), the type of place of residence: rural or urban; the cluster altitude, the household wealth index, the type of toilet facility, and the age and gender of the head of the household. In addition, the DHS program has now made available standardised files of the most commonly used geospatial covariates up to the year 2015, which can be linked to DHS datasets via the cluster ID [14]. Therefore, as no information regarding intestinal parasites (a known risk factor for anaemia [15]) was collected in the surveys used in this study, selected spatially indexed environmental covariates were considered as a proxy [16, 17]. Specifically, the cluster level average day land surface temperature (LST) and the cluster level average Enhanced Vegetation Index (EVI) for 2015. These explanatory variables considered in Fig. 1 were selected based on the literature as well as those available in the DHS and MIS data sets.

Furthermore, the spatial variation of childhood anaemia across the administrative levels of the countries was investigated. The administrative levels of each of the countries were chosen based on the levels for which public health decisions are made within each country. Accordingly, all 47 counties or districts for Kenya; 26 out of 28 districts for which data was available for Malawi; 176 out of 184 districts for which data was available for mainland Tanzania; and 121 out of 122 districts for which data was available for Uganda; a total of 370 districts were considered.

### Statistical methods

Non-spatial univariate logistic regression models were used to test for associations between each covariate and the child’s anaemia status. Covariates with associations that were significant at a 10% level were included in a hierarchical multivariable geoadditive logit model to control for the confounding effects of the covariates [18]. This formulation is a structured additive regression model that includes a spatial effect and is based on the generalised linear model (GLM) and generalised additive model (GAM) frameworks [19]. For this study, *Y*_*hijk*_ follows a Bernoulli distribution where *P*(*Y*_*hijk*_=1)=*π*_*hijk*_ is the probability that child *k* in household *j* within cluster *i* and district *h* is anaemic and *P*(*Y*_*hijk*_=0)=1−*π*_*hijk*_ is the probability that the child is not anaemic. The hierarchical geoadditive model is given by

1 $$ \begin{aligned} logit(\pi_{hijk}) &= \boldsymbol{x}'_{hijk} \boldsymbol{\beta} + f_{1}(z_{hijk1})+f_{2}(z_{hijk2})+ \ldots\\ &\quad+ f_{p}(z_{hijkp}) + f_{spat}(s_{h}) \end{aligned}  $$

where the left side of the Equation (1) is the logit link function and the right side is the geoadditive predictor. The parameter ***β*** is the vector of the linear fixed effects of the covariates that are modelled parametrically, and *f*_*r*_(.), *r*=1,…,*p*, are the unknown smooth functions that represent the non-linear effects of the continuous covariates which are modelled non-parametrically, thus Equation (1) is a semi-parametric model. The spatial effect of district *s*_*h*_ in which the child resides, *s*∈(1,…,370), is given by *f*_*spat*_(*s*_*h*_) which represents the effects of unobserved covariates that are not included in the model and also accounts for spatial autocorrelation [20]. This spatial effect may be partitioned into a spatially correlated (structured) and an uncorrelated (unstructured) effect as follows:

2 $$ f_{spat}(s_{h}) = f_{str}(s_{h}) + f_{unstr}(s_{h})  $$

The structured spatial effect *f*_*str*_(*s*_*h*_) accounts for the assumption that districts close in proximity are more likely to be correlated with regards to their outcomes. However, the unstructured spatial effect *f*_*unstr*_(*s*_*h*_) accounts for the spatial variation due to effects of unmeasured district-level factors that are not spatially related [21].

In this study, inference was fully Bayesian, hence all parameters and functions were treated as random variables. The fixed effect parameters in ***β*** were assigned vague Gaussian priors *N*(0,1000), with precision =0.001=1/variance. The Bayesian perspective of penalised splines (P-splines) was adopted for the unknown smooth functions *f*_*r*_ [22]. This approach assumes that the unknown functions can be approximated by a polynomial spline of degree *l* with equally spaced knots $z_{r}^{\min } = \zeta _{r0} < \zeta _{r1}< \ldots < \zeta _{rn_{r}-1}<\zeta _{rn_{r}}=z_{r}^{\max }$ which are within the domain of the covariate *z*_*r*_. The Bayesian spline can be written in terms of a linear combination of *M*_*r*_=*n*_*r*_+*l* polynomial B-spline basis functions, *B*_*rm*_, as follows

3 $$ f_{r}(z_{r}) = \sum\limits_{m=1}^{M_{r}} \alpha_{rm} B_{rm}(z_{r})  $$

Thus, $\boldsymbol {\alpha }_{r}=(\alpha _{r1}, \ldots, \alpha _{rM_{r}})'\phantom {\dot {i}\!}$ are unknown regression coefficients which are assigned first- or second-order random walk priors given by *α*_*rm*_=*α*_*r*,*m*−1_+*u*_*rm*_ and *α*_*rm*_=2*α*_*r*,*m*−1_−*α*_*r*,*m*−2_+*u*_*rm*_, respectively, with Gaussian errors $u_{rm} \sim N\left (0, \frac {1}{\tau ^{2}_{r}}\right)$ and diffuse priors *α*_*r*1_ or *α*_*r*1_ and *α*_*r*2_ as constants for initial values, respectively. The variance component $\tau ^{2}_{r}$ controls the smoothness of *f*_*r*_. In this study, second-order random walk smoothness priors and third degree splines were used.

For the structured spatial effect, *f*_*str*_(*s*_*h*_), intrinsic Gaussian Markov random field (IGMRF) priors specified by Besag et al. (1991) were used [23]. Two districts *s*_*h*_ and *s*_*i*_ are defined as neighbours if they share a common boundary. The spatial extension of random walk models leads to the conditional, spatially autoregressive specification:

4 $$ {}f_{str}(s_{h})|f_{str}(s_{i}),h\neq i \sim N\left(\frac{1}{n_{s_{h}}}\sum\limits_{s_{i} \in \delta_{s_{h}}} f_{str}(s_{i}), \frac{1}{n_{s_{h}}\tau_{str}^{2}}\right)  $$

where $n_{s_{h}}$ is the number of neighbours of district *s*_*h*_, and $s_{i} \in \delta _{s_{h}}\phantom {\dot {i}\!}$ denotes that district *s*_*i*_ is a neighbour of district *s*_*h*_. Therefore, the conditional mean of *f*_*str*_(*s*_*h*_) is an average of the function evaluations *f*_*str*_(*s*_*h*_) of neighbouring districts. Furthermore, the variance component $\tau _{str}^{2}$ controls the smoothness of the spatial effect and accounts for spatial variation between the districts, it is also used to capture the amount of variation explained by the spatial structure. The unstructured spatial effect *f*_*unstr*_(*s*_*h*_) was assigned i.i.d. Gaussian priors and specified as follows:

5 $$ f_{unstr}(s_{h}) \sim N\left(0, \frac{1}{\tau^{2}_{unstr}} \right)  $$

The variance components, *τ*^2^, of the random and spatial effects are unknown precision parameters that require estimation. Therefore, hyperpriors were assigned in a second stage of hierarchy. These hyperpriors are defined on a logarithmic scale and thus a log-gamma (*a*,*b*) distribution with hyper-parameters *a*=1 and *b*=0.001 was used. A sum-to-zero constraint was imposed on the non-linear and spatial effects to ensure model identifiability between the intercept and these effects.

Three types of models were fitted:Model 1: GLM model: Linear fixed effects of all variables, categorical and continuous. Model 2: GAM model: Linear fixed effects of categorical variables and some continuous variables, and non-linear effect of the child’s age in months.Model 3: Geoadditive Model: Model 2 with the inclusion of the spatial effects.

The posterior distributions of the parameters in the models were estimated using Integrated Nested Laplace Approximation (INLA) using the INLA package in R (http://www.r-inla.org/) [24]. INLA provides a faster alternative to Markov Chain Monte Carlo sampling (MCMC) and is a deterministic approach to approximate Bayesian inference [25]. The final geoadditive model was selected based on the Deviance Information Criteria (DIC), where the model with the smallest DIC was considered a better fit [26]. The sensitivity to the choice of the hyper-parameter values *a* and *b* was investigated by fitting the model with different hyper-parameter values [27]. However, the estimates had little sensitivity to these choices. QGIS 3.4 (https://qgis.org/en/site/index.html) was used to create maps displaying the posterior mean estimates of the spatial effects for the different districts of the countries.

## Results

### Sample characteristics

The final data set for this study consisted of 18247 children. Table S1 in Additional file 2 provides the sample sizes and percentage of anaemic children with the 95% confidence intervals according to the categorical predictors within each country and overall. These sample sizes and prevalence of anaemia were weighted to reflect the survey sampling weights. The observed prevalence of anaemia was lowest in Kenya at 36.4% (95% CI 34.3–38.5) with the other countries having much higher prevalences ranging from 53.0% to 61.9% (Uganda: 53.0%, 95% CI 51.3–54.5; Tanzania: 57.8%, 95% CI 56.6–59.0; Malawi: 61.9%, 95% CI 59.5–64.2). The overall prevalence of anaemia was 53.7% (95% CI 52.8–54.5). In all four countries there was a decrease in the observed prevalence of anaemia with an increase in mother’s education level and an improvement in toilet facilities. The observed prevalence of anaemia for those that tested positive for malaria according to the RDT (Kenya: 63.4%, 95% CI 57.2–69.2; Malawi: 77.7%, 95% CI 73.9–81.1; Tanzania: 82.7%, 95% CI 80.2–85.0; Uganda: 75.6%, 95% CI 73.1–77.9) was much higher than that of those who tested negative for malaria across all the countries (Kenya: 33.9%, 95% CI 31.1–36.8; Malawi: 52.8%, 95% CI 49.8–55.8; Tanzania: 53.5%, 95% CI 52.1–54.8; Uganda: 41.0%, 95% CI 41.0–45.0). Furthermore, children residing in rural areas had a higher observed prevalence of anaemia (Kenya: 39.2%, 95% CI 36.8–41.6; Malawi: 63.1%, 95% CI 60.4–65.7; Tanzania: 59.1%, 95% CI 57.7–60.5; Uganda: 54.1%, 95% CI 52.3–55.8) compared to those residing in urban areas in each of the countries (Kenya: 30.0%, 95% CI 26.3–34.0; Malawi: 53.9%, 95% CI 50.0–57.8; Tanzania: 53.9%, 95% CI 52.3–56.5; Uganda: 48.4%, 95% CI 44.4–52.5). Across all four countries, the observed prevalence of anaemia was fairly similar between children whose head of household was male or female.

### Results of the geoadditive model for anaemia

#### Model selection

Based on the non-spatial univariate logistic regression with 10% level of significance for inclusion, the only independent variable not entered into the multivariable model was the age of the head of household. The variance inflation factor (VIF) was used to check for collinearity among the remaining continuous independent variables and all variables had a VIF <4 and thus it was assumed that multicollinearity was not significantly present [28]. The non-linear effect of all continuous variables was investigated, however the only variable to display a significant non-linear effect on the log-odds of a child’s anaemia status was their age in months. Thus, this was the only non-linear effect considered in the models fitted, while the remaining independent variables were included as linear fixed effects. Table 1 presents the results of the DIC and effective number of parameters, *p*_*D*_, for each of the fitted models. Model 3 (Equation (1)) produced the lowest DIC, and thus the results of this study are based on this model, which includes both linear and non-linear effects as well as the spatial effects. It should be noted that the estimates of the fixed effects in the three models did not differ substantially, however the significance of the variables differed. Model 3, which accounted for spatial autocorrelation, resulted in two less statistically significant variables (EVI and LST) compared to models 1 and 2. Thus, failure to account for spatial autocorrelation would have produced misleading results.

**Table 1:** Model comparisons

	Model 1	Model 2	Model 3
DIC	22181.94	22086.94	21424.61
*p*_*D*_	16.01	22.96	263.45

#### Fixed effects

Table 2 displays the adjusted posterior odds ratio estimates (AOR) with their 95% credible intervals for the linear fixed effects included in the multivariable model. Female children had a significantly lower odds of anaemia compared to males [AOR = 0.873; 95% CrI 0.818–0.932]. Similarly, there was a significant decrease in the odds of anaemia with an increase in mother’s education, cluster altitude and household wealth index. Furthermore, a significantly lower odds of anaemia was suggested for children living in households with improved toilet facilities (PIT latrine and flush toilet). Children residing in urban areas had a lower odds of anaemia compared to those residing in rural areas, however these odds were not significantly different [AOR = 0.926; 95% CrI 0.835–1.027]. Children with a positive malaria RDT result had a significantly higher odds of anaemia compared to those who had a negative malaria RDT result [AOR = 4.401; 95% CrI 3.979–4.871], as did those children living in households with increasing number of residents [AOR = 1.019; 95% CrI 1.008–1.030]. While the odds of anaemia decreased with an increase in EVI [AOR = 0.987; 95% CrI 0.927–1.051] and increased with an increase in LST [AOR = 1.008; 95% CrI 0.994–1.022], these factors did not appear to be significantly associated with a child’s anaemia status.

**Table 2:** Adjusted posterior odds ratio estimates (AOR) and 95% credible intervals

Variable	AOR	95% Cred. Interval
Individual and Household Level		
*Gender (ref = Male)*		
Female	0.873 ^***∗***^	(0.818, 0.932)
*Malaria RDT Result (ref = Negative)*		
Positive	4.401 ^***∗***^	(3.979, 4.871)
*Household Size*	1.019 ^***∗***^	(1.008, 1.030)
*Type of Place of Residence (ref = Urban)*		
Rural	0.926	(0.835, 1.027)
*Mother’s Education Level (ref = No Education)*		
Primary	0.857 ^***∗***^	(0.773, 0.950)
Secondary and Higher	0.795 ^***∗***^	(0.694, 0.911)
Unknown	0.845 ^***∗***^	(0.742, 0.963)
*Gender of Household Head (ref = Male)*		
Female	1.003	(0.927, 1.086)
*Type of Toilet Facility (ref = No Facilities)*		
PIT Latrine	0.813 ^***∗***^	(0.723, 0.914)
Flush Toilet	0.749 ^***∗***^	(0.612, 0.916)
Other	0.711	(0.382, 1.325)
*Wealth Index*	0.858 ^***∗***^	(0.807, 0.911)
Cluster Level		
*Cluster Altitude (in 100 metres)*	0.974 ^***∗***^	(0.962, 0.987)
*EVI*	0.987	(0.927, 1.051)
*LST*	1.008	(0.994, 1.022)

^***∗***^significant at 5% level of significance

#### Non-linear and spatial effects

Table 3 provides the posterior mean and 95% credible interval for the smooth term variance components (the precisions) for the non-linear and spatial effects. The precision of an effect is the inverse of its variance. Thus, the larger the precision, the smaller the variance of the effect. The precision corresponding to the structured spatial effect (853.58) was much higher compared to that of the unstructured spatial effect (3.84), thus suggesting that the unstructured spatial effect was more dominant [29].

**Table 3:** Posterior mean and 95% credible interval (CrI) for the smooth term variance components

Variable	Mean	95% CrI
*Non-linear Effect*		
Child’s Age in Months ($\tau _{r}^{2}$)	1648.49	(485.52, 3938.21)
*Spatial Effect*		
Structured Spatial Effect ($\tau _{str}^{2}$)	853.58	(44.69, 3252.82)
Unstructured Spatial Effect ($\tau _{unstr}^{2}$)	3.84	(3.041, 4.78)

**Fig. 2:**
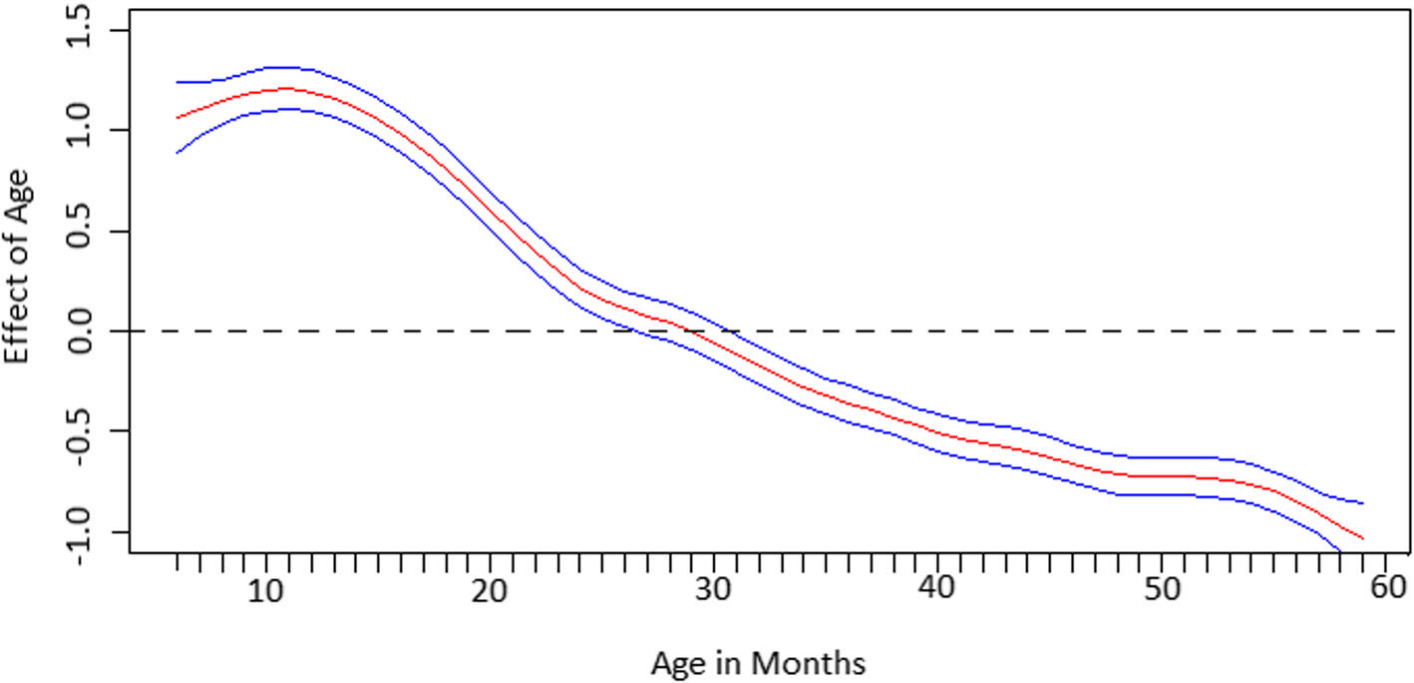
Estimated non-linear effects of child’s age in months on the log-odds of anaemia. The posterior mean together with the 95% credible intervals are shown

Figure 2 shows the non-linear effect that a child’s age in months has on the log-odds of being anaemic as well as the 95% credible interval. There was an increase in effect from 6 to 10 months, after which the effect declined. If a linear effect was used, it would have overestimated the effect of ages 30 to 50 months on anaemia.

**Fig. 3:**
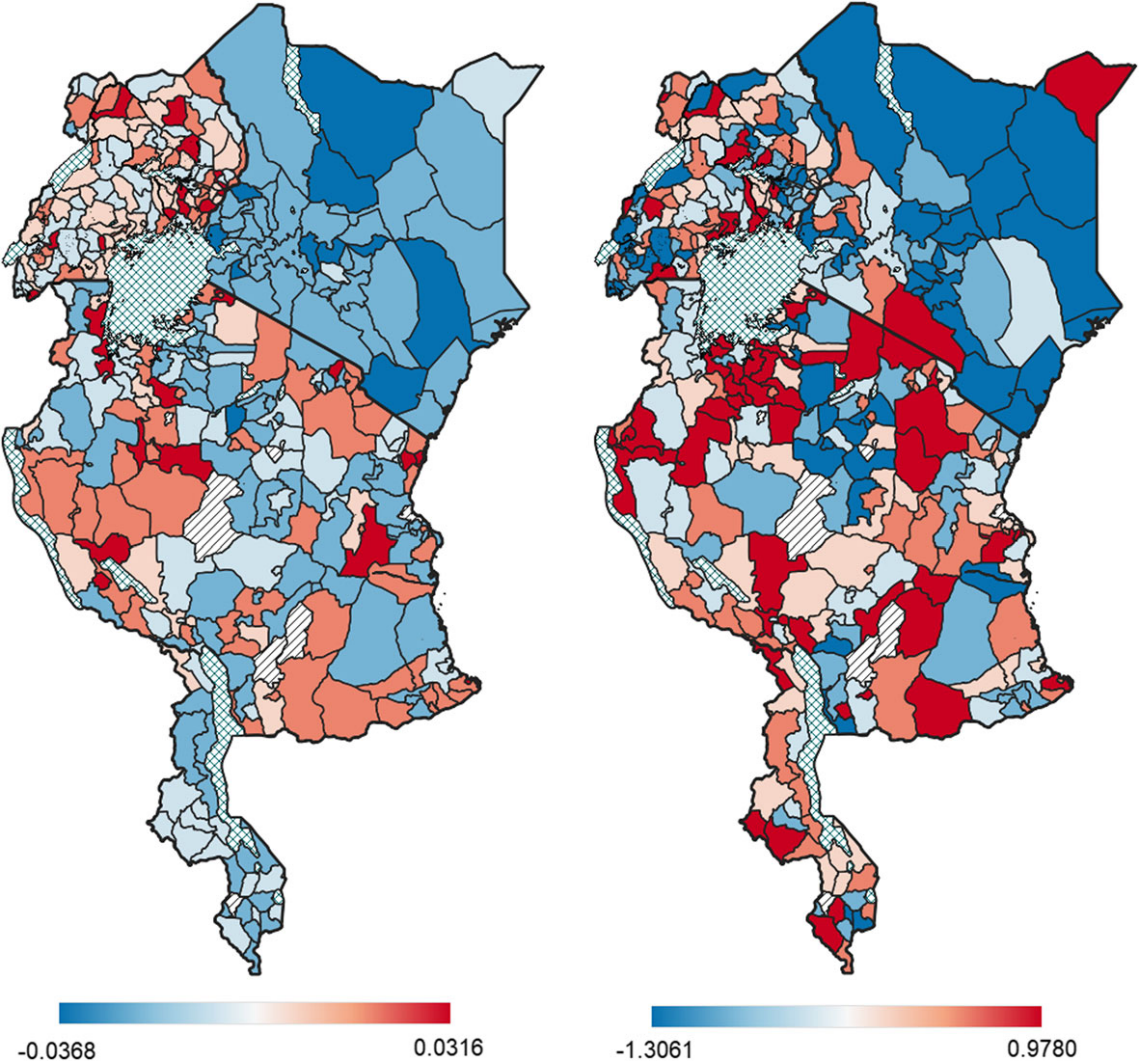
Estimated posterior means of the structured spatial effect (left) and the unstructured spatial effect (right) on the log-odds of anaemia (criss-cross pattern indicates water bodies; diagonal lines indicate districts with no data available) This figure is based on the results of this study and makes use of shapefiles freely available from the Spatial Data Repository (https://spatialdata.dhsprogram.com/boundaries)

Figure 3 displays the estimated means of the structured and unstructured spatial effects on the log-odds of anaemia, where the blue districts have a negative spatial effect and are therefore associated with a lower odds of anaemia, and the red districts have a positive spatial effect and are therefore associated with a higher odds of anaemia. The structured spatial effect, which ranged from −0.0368 to 0.0316, was weak in comparison to the unstructured spatial effect, which ranged from −1.3061 to 0.9780. Furthermore, the 95% CrI of the log-odds for the structured spatial effect in each district overlapped with the null of 0 (results not shown), thus the effects of spatially correlated factors contributing to childhood anaemia in all the districts were not statistically significant. However, 36 districts had a significantly positive unstructured spatial effect and 34 districts had a significantly negative unstructured spatial effect (see Figure S2 in Additional file 1).

## Discussion

This study utilised a hierarchical geoadditive logistic model to investigate the risk factors and spatial variation of anaemia in children aged 6 to 59 months in Kenya, Malawi, Tanzania and Uganda. This type of model allows one to assess and visualise the residual spatial effects on childhood anaemia while controlling for the effects of other covariates. Furthermore, it allows for the non-linear relationship of continuous covariates to be explored. In this study, incorporating the spatial effect in the model reduced the model’s DIC.

The results of this study confirm that of other studies, where girls are less at risk of anaemia, and a child’s risk decreases with an increase in mother’s education level and wealth [21, 30–32]. This may be due to more educated individuals being more aware and having more of an understanding of health related issues. Similarly, this could be said of individuals with more wealth. However, a lack of wealth also restricts an individual’s ability to access good health care and nutritional food sources. Having malaria was associated with a significantly higher risk of anaemia, thus suggesting much of the burden of childhood anaemia in these countries is contributed by malaria. The type of toilet facilities was significantly associated with a child’s anaemia status. Poor sanitation is a known risk factor of the intestinal parasite hookworm which causes anaemia in infected children [33]. While a study by Soares Magalhães and Clements (2011) [34] found environmental factors LST and the normalized difference vegetation index (NDVI) to be significantly associated with an increased risk of anaemia in preschool-age children, the environmental factors LST and EVI considered in this study were not found to be significantly associated with anaemia. However, such environmental factors, especially EVI, are known to be highly correlated with malaria, and thus the inclusion of the child’s malaria status may account for much of the effects that these environmental factors have on childhood anaemia [35, 36].

The non-linear effect of the child’s age on anaemia displayed an increase from 6 to 10 months, after which the effect declined. Multiple factors could be contributing to this increased risk of anaemia in children aged 6 to 10 months. Either these children are not receiving adequate nutrients or they are experiencing a decrease in their Hb concentrations due to other factors. Infants are born with a reserve of iron which is responsible for growth and protection from iron deficiency in the first 4 to 6 months of life [37]. After 6 months of age, the iron store is depleted, and thus it is common for milk supplements to be introduced into a child’s diet to complement breastfeeding as breast milk alone may not provide sufficient iron to meet the demand of the rapid growth experienced in children during this period [30, 38]. However, safe complementary feeding in children from 6 months is not always practised, where the feeding of unmodified cow’s milk in children less than 12 months of age is common in some SSA countries despite evidence of increased risk of iron-deficiency anaemia and other adverse health outcomes [39, 40]. Wijndaele et al. (2009) [41] found that low maternal education and low socio-economic status are associated with feeding of unmodified cow’s milk in children less than a year old. In addition, malaria in mothers may also be a contributing factor to the increased risk of anaemia in children aged 6 to 10 months, where White (2018) [7] states that the effects of maternal anaemia due to malaria can cause a physiological decline in Hb concentrations in infants from birth up to 9 months of age, after which there is a slow but steady rise in Hb concentrations. Other studies on young children from Kenya, Malawi, Tanzania and Uganda have also reported similar patterns of decreased Hb concentrations in children less than 10 months of age [21, 42–45].

The benefit of focusing on more than one country at a time is that one is able to consider whether factors that transcend boundaries are significantly contributing to childhood anaemia, such as environmental and geographical factors. This study revealed that the structured spatially correlated effect was fairly weak in comparison to the unstructured spatial effect, suggesting that the contribution that a particular district has on the risk of anaemia is not similar among neighbouring districts. This is an indication that environmental and geographical factors that transcend boundaries of the districts may not play a significant role in childhood anaemia. With the unstructured spatial effect being more prominent in this study, it can be concluded that there are unmeasured district-specific factors that are not spatially structured (that are not correlated with that of neighbouring districts) contributing to childhood anaemia. In addition, there was a distinct pattern of variation in the spatial effects across the districts within each country, except for Kenya which was fairly homogeneous in both types of spatial effects. Kenya has made substantial progress in the reduction of malaria, however this has resulted in a heterogeneous risk of malaria across the country [46]. Thus, the homogeneous results of the spatial effects on childhood anaemia in Kenya could be due to the strong correlation between malaria and anaemia in the country, which is being accounted for by the inclusion of the child’s malaria status. However, the spatial effects in Uganda, Tanzania and Malawi remain heterogeneous even after controlling for the child’s malaria status, thus there are other significant drivers of childhood anaemia in these countries. On the whole, the spatial effects do not appear to transcend the borders between the countries as the pattern of effects differed around the borders, barring Longido district in Tanzania and Kajiado county in Kenya which share a border. This indicates that there are country-specific factors contributing to anaemia in children. Such factors may include the cost and quality of health care, and the cost of living, which can vary considerably between and within countries, the effects of which have been known to contribute to the spatial variation of other childhood diseases [20].

A limitation of this study is that it is based on secondary data from cross-sectional surveys, therefore a causal relationship cannot be established. In addition, no information on iron levels in the children was available, however iron deficiency plays a major role in childhood anaemia [47]. Furthermore, while this study could not assess the contribution of intestinal parasites to the burden of anaemia in children directly, proxies for this factor was used instead. The strength of this study lies in utilising individual level malaria RDT results rather than estimates or indicators of malaria.

## Conclusion

While the WHO recommends daily iron supplementation in infants and young children aged 6 to 59 months living in settings where the prevalence of anaemia is 40% or higher in these age groups [48], this should be accompanied by programs that create awareness about the causes and consequences of anaemia in children, especially targeting the parents of children in the younger age group (6 to 10 months), regardless of the prevalence of anaemia in this age group. Furthermore, programs that ensure the introduction of safe and adequate complementary foods in a child’s diet from the age of 6 months should be considered. These types of programs would be beneficial as these children are more susceptible to anaemia due to the rapid growth during that stage of their lives.

Anaemia control measures in Malawi, Tanzania and Uganda need to account for the spatial heterogeneity that is evident in these countries, as well as take into consideration the potential factors and type of factors (local or otherwise) contributing to the spatial heterogeneity. Efforts in assessing the local district-specific causes of childhood anaemia within each country should be focused on.

## Supplementary information

**Additional file 1** Figure S1 (Location of study areas) and Figure S2 (Significance of the unstructured spatial effect).

**Additional file 2** Table S1 (weighted sample sizes and observed prevalence of anaemia within each country and overall).

## Abbreviations

95% CrI: 95% credible intervals
AOR: Adjusted odds ratio
DHS: Demographic and Health Survey
DIC: Deviance Information Criteria
EVI: Enhanced vegetation index
GAM: Generalised additive model
GLM: Generalised linear model
Hb: Haemoglobin
HIC: High income countries
LMIC: Low and middle income countries
LST: Land surface temperature
MIS: Malaria Indicator Survey
SSA: sub-Saharan Africa
