# Supplementary material for: Investigating the spatial variation and risk factors of childhood anaemia in four sub-Saharan African countries
Source: BMC Public Health. 2020 Jan 29;20:126. doi: 10.1186/s12889-020-8189-8 (PMC6990548; doi:10.1186/s12889-020-8189-8)
Supplement: Supplementary file 1 — Additional file 1 Figure S1 (Location of study areas) and Figure S2 (Significance of the unstructured spatial effect). [file 12889_2020_8189_MOESM1_ESM.pdf]

Additional File 1: Investigating the spatial variation and risk factors of childhood anaemia in 4 sub-Saharan African countries

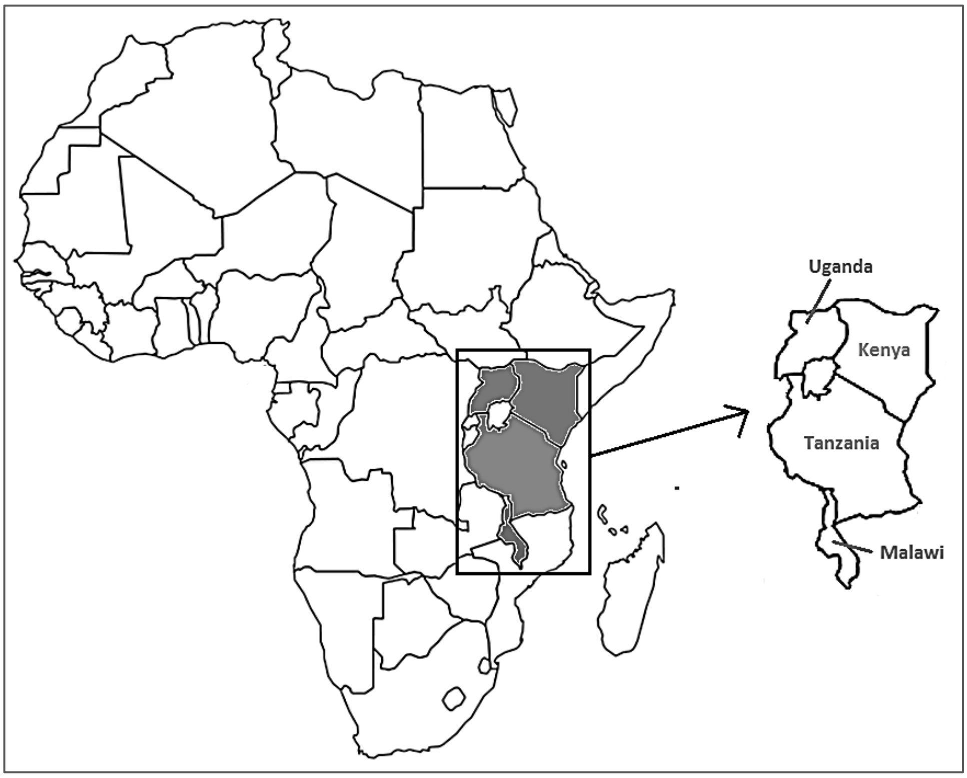

Figure S1: **Location of study areas**

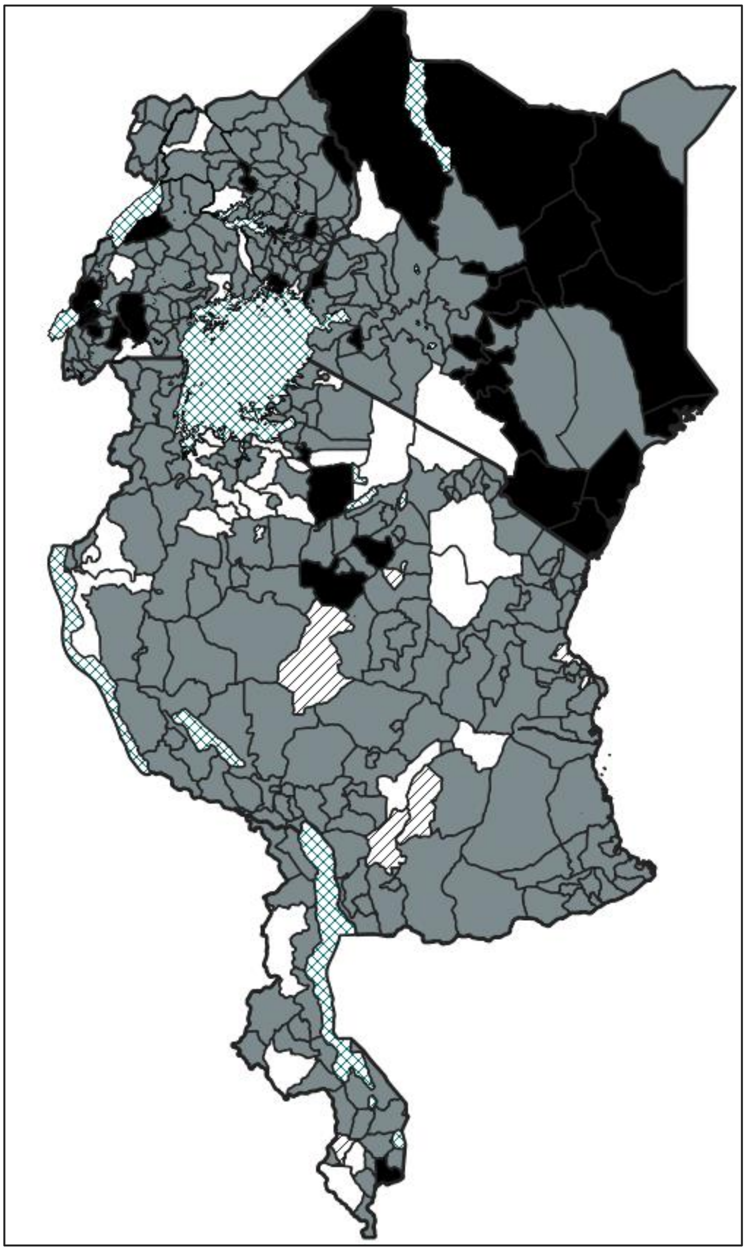

Figure S2: **Significance of the unstructured spatial effect. Black districts – significant negative effect, grey districts - no significance, white districts- significant positive effect (criss-cross pattern indicates waterbodies; diagonal lines indicate districts with no available data).** This figure is based on the results of this study and makes use of shapefiles freely available from the Spatial Data Repository (https://spatialdata.dhsprogram.com/boundaries)
