# Supplementary material for: Investigating the spatial variation and risk factors of childhood anaemia in four sub-Saharan African countries
Source: BMC Public Health. 2020 Jan 29;20:126. doi: 10.1186/s12889-020-8189-8 (PMC6990548; doi:10.1186/s12889-020-8189-8)
Supplement: Supplementary file 2 — Additional file 2 Table S1 (weighted sample sizes and observed prevalence of anaemia within each country and overall). [file 12889_2020_8189_MOESM2_ESM.pdf]

Table I: Sample size (n) and percentage of anaemic children (95% confidence interval) according to the categorical predictors within each country based on sampling weights

**Characteristics**	**Country**								**Overall**	
	**Kenya**		**Malawi**		**Tanzania**		**Uganda**			
	n	**% Anaemic (95% CI)**	n	**% Anaemic (95% CI)**	n	**% Anaemic (95% CI)**	n	**% Anaemic (95% CI)**	n	**% Anaemic (95% CI)**
Gender										
Male	1566	38.5 (35.2, 41.2)	1256	61.1 (57.7, 64.4)	4349	59.6 (57.8, 61.3)	2351	53.5 (51.2, 55.9)	9522	54.8 (53.6, 56.0)
Female	1513	34.5 (31.8, 37.4)	1235	62.6 (59.2, 65.9)	4299	56.0 (54.3, 57.8)	2336	52.4 (50.1, 54.7)	9383	52.5 (51.3, 53.7)
Malaria										
Positive	277	63.4 (57.2, 69.2)	906	77.7 (73.9, 81.1)	1284	82.7 (80.2, 85.0)	1433	75.6 (73.1, 77.9)	3900	77.6 (76.0, 79.1)
Negative	2802	33.9 (31.1, 36.8)	1585	52.8 (49.8, 55.8)	7364	53.5 (52.1, 54.8)	3254	43.0 (41.0, 45.0)	15005	47.4 (46.5, 48.4)
Type of Place of Residence										
Urban	934	30.0 (26.3, 34.0)	328	53.9 (50.0, 57.8)	2168	53.9 (51.3, 56.5)	900	48.4 (44.4, 52.5)	4330	47.6 (45.8, 49.5)
Rural	2145	39.2 (36.8, 41.6)	2163	63.1 (60.4, 65.7)	6480	59.1 (57.7, 60.5)	3787	54.1 (52.3, 55.8)	14575	55.4 (54.5, 56.4)
Mother's Education Level										
No Education	359	47.2 (42.3, 52.3)	327	68.9 (61.8, 75.2)	1692	66.4 (63.7, 69.0)	463	61.6 (56.7, 66.3)	2841	63.5 (61.4, 65.5)
Primary	1343	38.1 (35.0, 41.3)	1480	64.3 (61.2, 67.4)	5037	56.9 (55.3, 58.5)	2441	54.3 (52.1, 56.4)	10301	54.9 (53.8, 56.0)
Secondary or Higher	859	32.8 (28.7, 37.2)	367	52.7 (47.3, 58.1)	968	54.4 (50.6, 58.2)	1062	49.6 (45.9, 53.4)	3256	47 (44.8, 49.1)
Unknown	518	30.3 (26.0, 34.9)	317	53.6 (46.7, 60.3)	951	50.7 (46.9, 54.5)	721	48.1 (43.9, 52.3)	2507	46.1 (43.8, 48.4)
Gender of Household Head										
Male	2100	36.5 (34.1, 39.0)	1921	61.0 (58.3, 63.6)	7020	57.8 (56.5, 59.2)	3373	53.1 (51.2, 55.1)	14414	54.0 (53.1, 55.0)
Female	979	36.1 (32.4, 40.0)	570	64.8 (59.8, 69.6)	1628	57.5 (54.2, 60.8)	1314	52.6 (49.5, 55.6)	4491	52.4 (50.6, 54.2)
Type of Toilet Facility										
No Toilet Facility	487	49.3 (44.8, 53.8)	169	68.9 (58.8, 77.4)	1118	70.1 (66.9, 73.0)	375	66.9 (61.9, 71.5)	2149	64.7 (62.4, 66.9)
PIT Latrine	2325	35.0 (32.7, 37.3)	2279	62.0 (59.5, 64.4)	6480	56.9 (55.5, 58.3)	4196	52.0 (50.2, 53.7)	15280	53.0 (52.0, 53.9)
Flush Toilet	266	25.0 (17.5, 34.3)	43	29.7 (20.7, 40.6)	1022	50.7 (46.8, 54.5)	97	42.1 (30.0, 55.1)	1428	44.6 (41.3, 48.0)
Other	2	50.0 (5.9, 94.1)	0	0	28	43.5 (26.2, 62.6)	18	61.3 (35.7, 81.9)	48	49.9 (35.1, 64.7)
**Total**	**3079**	**36.4 (34.3, 38.5)**	**2491**	**61.9 (59.5, 64.2)**	**8648**	**57.8 (56.6, 59.0)**	**4687**	**53.0 (51.3, 54.6)**	**18905**	**53.7 (52.8, 54.5)**
